# Exploring Socioeconomic Status as a Global Determinant of COVID-19 Prevalence, Using Exploratory Data Analytic and Supervised Machine Learning Techniques: Algorithm Development and Validation Study

**DOI:** 10.2196/35114

**Published:** 2022-09-27

**Authors:** Luke Winston, Michael McCann, George Onofrei

**Affiliations:** 1 Department of Computing Atlantic Technological University Letterkenny Ireland; 2 Department of Business Atlantic Technological University Letterkenny Ireland

**Keywords:** COVID-19, machine learning, data analysis, epidemiology, human development index

## Abstract

**Background:**

The COVID-19 pandemic represents the most unprecedented global challenge in recent times. As the global community attempts to manage the pandemic in the long term, it is pivotal to understand what factors drive prevalence rates and to predict the future trajectory of the virus.

**Objective:**

This study had 2 objectives. First, it tested the statistical relationship between socioeconomic status and COVID-19 prevalence. Second, it used machine learning techniques to predict cumulative COVID-19 cases in a multicountry sample of 182 countries. Taken together, these objectives will shed light on socioeconomic status as a global risk factor of the COVID-19 pandemic.

**Methods:**

This research used exploratory data analysis and supervised machine learning methods. Exploratory analysis included variable distribution, variable correlations, and outlier detection. Following this, the following 3 supervised regression techniques were applied: linear regression, random forest, and adaptive boosting (AdaBoost). Results were evaluated using k-fold cross-validation and subsequently compared to analyze algorithmic suitability. The analysis involved 2 models. First, the algorithms were trained to predict 2021 COVID-19 prevalence using only 2020 reported case data. Following this, socioeconomic indicators were added as features and the algorithms were trained again. The Human Development Index (HDI) metrics of life expectancy, mean years of schooling, expected years of schooling, and gross national income were used to approximate socioeconomic status.

**Results:**

All variables correlated positively with the 2021 COVID-19 prevalence, with R^2^ values ranging from 0.55 to 0.85. Using socioeconomic indicators, COVID-19 prevalence was predicted with a reasonable degree of accuracy. Using 2020 reported case rates as a lone predictor to predict 2021 prevalence rates, the average predictive accuracy of the algorithms was low (R^2^=0.543). When socioeconomic indicators were added alongside 2020 prevalence rates as features, the average predictive performance improved considerably (R^2^=0.721) and all error statistics decreased. Thus, adding socioeconomic indicators alongside 2020 reported case data optimized the prediction of COVID-19 prevalence to a considerable degree. Linear regression was the strongest learner with R^2^=0.693 on the first model and R^2^=0.763 on the second model, followed by random forest (0.481 and 0.722) and AdaBoost (0.454 and 0.679). Following this, the second model was retrained using a selection of additional COVID-19 risk factors (population density, median age, and vaccination uptake) instead of the HDI metrics. However, average accuracy dropped to 0.649, which highlights the value of socioeconomic status as a predictor of COVID-19 cases in the chosen sample.

**Conclusions:**

The results show that socioeconomic status is an important variable to consider in future epidemiological modeling, and highlights the reality of the COVID-19 pandemic as a social phenomenon and a health care phenomenon. This paper also puts forward new considerations about the application of statistical and machine learning techniques to understand and combat the COVID-19 pandemic.

## Introduction

### Background

The COVID-19 pandemic represents the most unprecedented global challenge in recent times. Originally identified in the city of Wuhan, China, the SARS-CoV-2 virus spread across the world, and the situation escalated into an international emergency. Despite widescale containment efforts in 2020, as well as the largest vaccine rollout in history [1], the pandemic continued to challenge the global community in 2021. Research is being conducted to analyze the trajectory of the virus, and to understand why particular populations or countries have been more severely impacted than others [2,3]. This has been supported by increases in data availability, which has enabled researchers to investigate a large range of potential COVID-19 risk factors. These risk factors can be categorized as clinical or nonclinical. Clinical risk factors include obesity [4-6], diabetes [7,8], and smoking [9]. Examples of nonclinical risk factors are cultural differences [10], government containment measures [11], vaccination attitudes [12], and socioeconomic status [13-15].

This paper focuses on the nonclinical risk factor of socioeconomic status as a determinant of COVID-19 prevalence. To provide a reliable empirical metric for socioeconomic status, the Human Development Index (HDI) of the United Nations Development Programme (UNDP) was selected. The HDI calculates the overall socioeconomic status or “well-being” of inhabitants in a country by aggregating its life expectancy, education, and per capita income metrics [16]. It has been applied successfully in previous epidemiological research to map prevalence rates of various diseases [17-20]. Despite its popularity in statistical analysis, the HDI has not yet been widely applied in machine learning COVID-19 modeling. This presents an opportunity to apply statistical and machine learning techniques to examine whether the HDI can be used to accurately predict prevalence rates of COVID-19.

### Related Work

#### Socioeconomic Status in Health Research

Pandemics are as much a social problem as a health care problem [21]. As such, socioeconomic status is an important determinant to consider in pandemic research. The term socioeconomic status is an umbrella term used to describe empirically measurable social or economic factors, such as social class, education, income, and health [22,23]. These factors are applied in a variety of ways to investigate or control their effects on given outcomes, such as health outcomes, and have consistently been found to be statistically significant [24-26]. In terms of health outcomes, higher socioeconomic status has typically been associated with better health. Conversely, lower socioeconomic status is associated with poorer health outcomes [27]. In the literature, lower socioeconomic status has been associated with higher rates of illnesses, such as osteoarthritis, chronic diseases, hypertension, and cervical cancer [28,29].

In relation to COVID-19, socioeconomic status has also been associated with higher prevalence and more severe outcomes. In the United States, the Distressed Communities Index has been used to analyze the impact of socioeconomic status on COVID cases and mortality [30]. Results from this study indicated that lower education and racial differences were associated with poorer COVID-19 outcomes. Another study argued that lower socioeconomic populations are more likely to live in overcrowded accommodation and have less access to outdoor space, making them more vulnerable to COVID-19 infection [31]. Evidently, socioeconomic status is an important determinant of COVID-19 outcomes, which can uncover how the virus affects particular populations.

#### HDI

The HDI is a composite measure of overall socioeconomic status at the national level, which is annually calculated by the UNDP. The HDI indices include life expectancy, expected years of schooling, mean years of schooling, and gross national income (GNI). Calculating a country’s HDI for a given year requires 2 steps. First, values from each of the 4 indices are normalized to an index value between 0 and 1. Maximum and minimum limits for each metric are set by the UNDP. Using the actual value, maximum value, and minimum value, the dimension index can be calculated with the following formula:

Dimension index = (actual value − minimum value) / (maximum value − minimum value)

Second, once each individual dimension has been calculated, the equally weighted mean is calculated to provide the overall HDI score of a country [32].

The HDI has been used in health research to analyze both the prevalence rates and mortality rates of specific diseases, which helps to identify disparities in terms of outcome within a country or between countries. It has been applied to understand a range of epidemiological research problems, such as malaria [17], various cancer distributions [19,33,34], hypertension [20], *Blastocystis* parasites [35], and dental health [36]. To provide a specific example, research investigating the relationship between the HDI and thyroid cancer suggested that although higher HDI countries have a higher prevalence of the disease, lower HDI countries have higher mortality rates [34].

The HDI has also been applied to analyze the ongoing COVID-19 pandemic, generating important insights about the disproportionate impact of the pandemic cross-nationally. For example, a study analyzing the HDI and COVID-19 mortality reported that countries with high HDI scores recorded higher COVID-19 mortality rates [13]. Another study reported significant correlations between the HDI scores of 166 countries and their confirmed cases on March 27, 2020 [14]. Elsewhere, a study focusing on municipal differences in COVID-19 impact in Brazil (using a recalibrated index to analyze municipal differences rather than national differences) found that municipalities with high HDI scores had the highest COVID-19 incidence rate and mortality per 100,000 population as of May 2020 [15]. The index has therefore been recognized as a valuable framework in COVID-19 research.

#### Multicountry COVID-19 Research

Multicountry COVID-19 research is important for the following 2 reasons: (1) the ability to identify country-specific points of interest, and (2) the ability to uncover common trends or risk factors across countries. In a study of lockdown-associated mental health problems in Egypt, Pakistan, India, Ghana, and the Philippines, it was reported that although lockdowns negatively affected the mental health of respondents in each country, they did so in different ways. For example, respondents from the Philippines coped with lockdowns by increasing self-destructive behaviors, while those from Pakistan sought comfort in religion. Respondents from the 3 remaining countries tended to accept the lockdowns [37]. A similar study in a larger sample of 101 countries analyzed the loneliness and social isolation associated with the pandemic [38]. Other studies have been conducted to analyze cross-national vaccination attitudes [39], the success of containment measures [11,40], and cultural behaviors that impacted cross-national COVID-19 mortality rates [10]. Therefore, multicountry COVID-19 research helps to identify “global risk factors” relating to the pandemic, subsequently aiding evidence-based public health interventions [38]. It also opens up new research questions as to why certain populations behaved or were impacted a certain way during the pandemic.

#### Modeling Outbreaks Using Machine Learning

When modeling outbreaks, a popular method in epidemiology is the susceptible, infected, recovered (SIR) approach. The SIR approach simplifies the transmission dynamics of infectious diseases by dividing the population into groupings of susceptible, infected, and recovered individuals and analyzes the interaction between these groups over the course of an outbreak. This method has also been deployed to analyze the COVID-19 pandemic [41,42]. However, SIR modeling assumes that complete herd immunity is possible through infection [43], which limits its efficacy in COVID-19 research. It is not yet understood if COVID-19 herd immunity is achievable due to the complex nature of the virus, the questionable long-term efficacy of available vaccines, the emergence of new variants, and the cases of reinfection [44]. Subsequently, the predictive benefits of machine learning may yield better results in relation to this pandemic.

Advancements in machine learning have enabled epidemiological researchers to use a robust data-driven approach facilitated by high-precision algorithms. This has helped to process ever-increasing volumes of data, and to analyze a wider range of factors that impact patient health outcomes [45,46]. For example, naïve Bayes, logistic regression, random forest, and artificial neural network models have been developed to predict hypotension in patients after receiving an anesthetic [47]. Elsewhere, gated recurrent unit neural networks have been designed to identify individuals at risk of in-hospital mortality. This model allows practitioners to map the probability of death longitudinally, and to provide targeted interventions based on the model predictions [48].

Another advantage of machine learning in epidemiology is that it can predict and map disease occurrences and health outcomes in situations where data are limited [49]. Specifically, boosted regression tree models have been used to analyze environmental factors that affect the transmission of diseases, such as dengue fever, Ebola, Crimean-Congo hemorrhagic fever, and Zika virus [50-53]. Another type of machine learning model, the Ensemble Adjustment Kalman Filter, has been used to forecast seasonal outbreaks of influenza [54]. Additionally, several retrospective forecasting studies have been conducted to reconstruct past pandemics, including Ebola, West Nile Virus, and Respiratory Syncytial Virus, by mapping their transmission patterns [55-57].

Regarding COVID-19, epidemiological research using machine learning is emerging in the literature at pace. Generally, studies have involved the design of one or more machine learning models to predict COVID-19 case prevalence [11,58,59], severity [60,61], and mortality/risk of mortality [62,63]. In 1 study, 5 non–time series supervised learning models using random forest and AdaBoost regression were trained to predict the confirmed infection growth (the 14-day growth of the cumulative number of reported COVID-19 cases) of COVID-19 in 114 countries, using nonpharmaceutical containment measures and cultural dimensions as features. Results indicated that confirmed infection growth was predicted to a considerable degree with moderate to high R^2^ values (>0.50) [11]. Lastly, a systematic review of machine learning techniques in the prediction of COVID-19 cases found that R^2^ values ranged between 0.64 and 1, suggesting that machine learning is a highly valuable method for predicting COVID-19 prevalence, which could support policy makers in shaping future interventions [64].

### Description of the Study

This study analyzed the statistical relationship between HDI scores and cumulative COVID-19 cases (total recorded cases up to December 31, 2021) in a sample of 182 countries. It then attempted to predict 2021 COVID-19 cumulative cases in the sample using the previous year’s cumulative cases (total recorded cases up to December 31, 2020) and HDI scores. Cumulative cases per million of the population was selected as it provides the number of reported infections proportionate to the population size. Crude rate metrics, such as cases per million, are the most effective for multicountry samples [65]. For example, Afghanistan and Albania reported a similar absolute number of COVID-19 cases in 2020, with values of 51,526 and 58,316, respectively. However, Afghanistan’s cases per million was 1324, while Albania’s was 20,264. This shows the viral prevalence relative to both populations and indicates that Albania actually had higher case rates in 2020.

To measure socioeconomic status, the HDI indices of life expectancy, expected years of schooling, mean years of schooling, and GNI were used. For the purposes of this study, individual metrics were selected rather than the aggregated HDI value. This approach was used because aggregation can lose important information in the data, which can lead to less accurate predictions [66].

Two predictive models were designed using the open-source integrated development environment Jupyter Notebook, which is compatible with Python programming language. Each model was trained using the following 3 supervised learning regression algorithms: basic linear regression, random forest, and AdaBoost. All algorithms were evaluated using k-fold cross-validation and then compared by calculating their R^2^ scores and error statistics. The first model attempted to predict 2021 COVID-19 prevalence using 2020 case numbers to establish a baseline for the performance of the second model. The second model included 2020 case numbers and each country’s life expectancy, expected years of schooling, mean years of schooling, and GNI metrics. Due to the uneven progress of the pandemic on a country-by-country basis, this study focused on cross-sectional data rather than time-series data. All data for this study are secondary and publicly available, highlighting the commendable global effort to collect and share data concerning the pandemic.

## Methods

### Data Preprocessing

COVID-19 case data were downloaded from the COVID-19 OurWorldInData database [65], which in turn retrieves data from the John Hopkins Center for Systems Science and Engineering Data Repository. The OurWorldInData database contains comprehensive COVID-19 metrics for 190 countries, including infection rates, hospitalization numbers, mortality rates, and vaccination uptake figures. Data are uploaded daily, which allows users to track the evolution of the pandemic with up-to-date statistics. This research required each country’s “cases per million” figure for December 31, 2020, and the same metric for December 30, 2021. HDI data were extracted from the 2020 Human Development Report Data Center [67]. The report provides each country’s overall HDI score and the score for each individual metric.

These data sets were combined so that each observation (country) contained the following metrics: (1) life expectancy, (2) expected years of schooling, (3) mean years of schooling, (4) GNI, (5) COVID-19 cases per million in 2020 (January 1-December 31), and (6) COVID-19 cases per million in 2021 (January 1-December 31).

Countries with missing data were omitted; therefore, the final data set contained data for 182 countries. It was then imported to Jupyter and converted into dataframe format (see Table 1) to begin analysis.

Following this, exploratory data analysis was conducted to explore the distribution of the data and the statistical relationships between the variables. A data scaling method was then selected depending on the distribution of the data. Data scaling is important in machine learning modeling as it prevents measurement differences from negatively affecting the final results [68]. The interquartile range was then calculated to identify outliers in the target variable (2021 COVID-19 cases).

**Figure 1:**
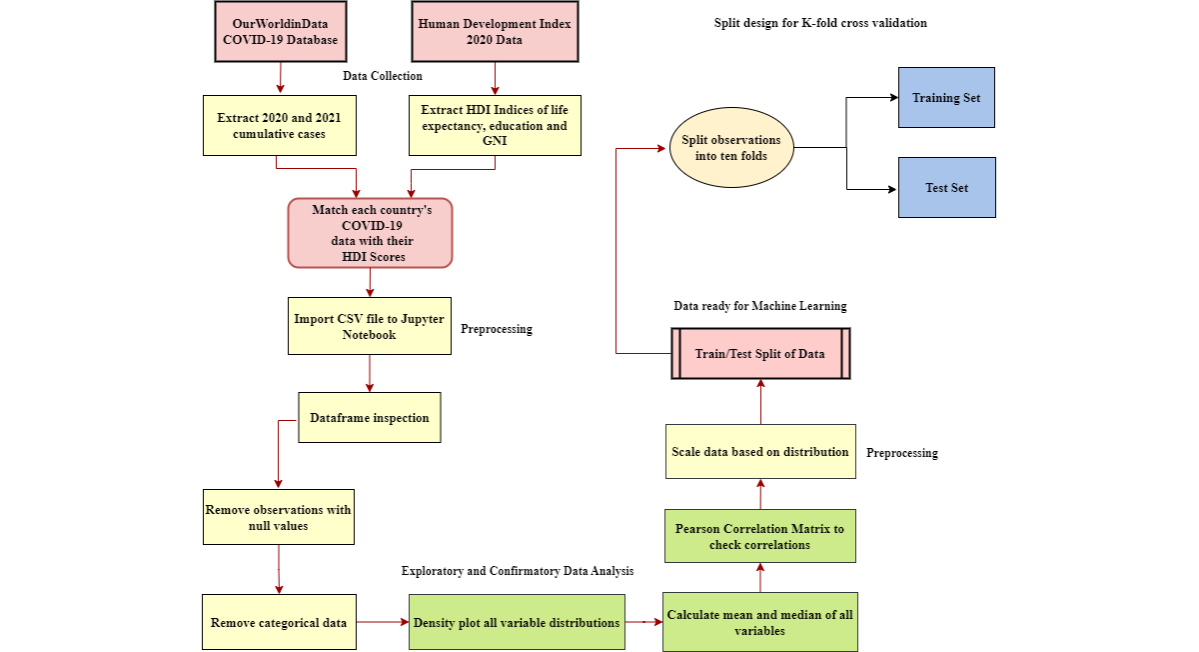
A flowchart illustrating the data pipeline, from the collection of COVID-19 and Human Development Index (HDI) data to the cross-validation training and testing process. In addition to designing the predictive models, exploratory data analysis was also conducted to identify trends in the data set. GNI: gross national income.

Figure 1 summarizes the workflow for this study, from data preprocessing to model design and exploratory data analysis.

**Table 1:** Sample of the data set using Human Development Index metrics and COVID-19 cases.

Country	Life expectancy	Expected years of schooling	Mean years of schooling	Gross national income per capita (US$)	Cases 2020 (per million)	Cases 2021 (per million)
Afghanistan	64.8	10.2	3.9	2239	1323.612	3968.427
Albania	78.6	14.7	10.1	13,998	20,264.091	73,173.975
Algeria	76.9	14.6	8.0	11,174	2271.554	4895.753
Andorra	81.9	13.3	10.5	56,000	104,173.947	306,900.742
Angola	61.2	11.8	5.2	6104	534.073	2404.489

### Machine Learning Algorithm Selection

Supervised machine learning models are trained to make predictions by learning from a data set where the value of the output (dependent variable) is known for each observation. Supervised machine learning produces decisions or “outputs” based on input data during the training process. Implementing different supervised algorithms on a set of data allows for the results to be compared and for the best fitting model to be identified [69,70]. Evaluating a supervised learning model requires robust validation measures [71]. These can be calculated using a variety of accuracy and error metrics, such as the coefficient of determination (R^2^), mean absolute error (MAE), mean squared error (MSE), root mean squared error (RMSE), or max error. This research compared the performances of linear regression, random forest, and AdaBoost supervised techniques.

#### Linear Regression

Linear regression is one of the most common machine learning algorithms [72]. Regression in machine learning differs from traditional statistical regression as it partitions the data set into a training set and a test set. Using the input and output data from the training set, algorithms attempt to predict output data in the test set using input data only. This process indicates how accurately a model can make predictions on new data. Linear regression is calculated as follows:

*y* = *a_0_* + *a_1_x* + *ε*

where *y* is the target variable (output), *x* is the predictor variable (input), *a_0_* is the intercept, *a_1_* is the coefficient, and *ε* is random error.

#### Random Forest

Random forest is an ensemble of decision tree algorithms that can be used for either classification or regression problems. It is based on the concept of bagging or bootstrap aggregation, which creates an ensemble of learner trees [73]. Each learner tree (*K*) is trained on separate samples drawn from the original data set (input vector *x*), and the overall prediction is obtained by calculating the mean of *K* regression trees as follows:



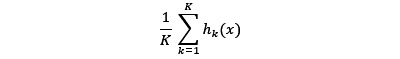



Random forest is beneficial for reducing model variance compared to individual decision trees. It also helps to prevent model overfitting (when a model fits too closely to training data and poorly to test data) [74].

#### AdaBoost

AdaBoost or adaptive boosting is a sequential ensemble technique that is based on the principle of developing several weak learners using different training subsets drawn randomly from the original training data set. Using this technique, the training algorithm begins with 1 decision tree, identifies the observations with the highest error, and adds more weight to these. The weights are recalculated after every iteration so that incorrectly classified observations by the previous decision tree receive higher weights [75]. Using Python programming language, the number of trees that the algorithm will deploy can be chosen, with the default set at 50 iterations.

### Model Design and Evaluation

Two feature models were created (Feature Model 1 and Feature Model 2). Feature Model 1 was trained to predict 2021 COVID-19 prevalence using 2020 cases only. Feature Model 2 was trained to predict 2021 COVID-19 prevalence using 2020 case data as well as life expectancy, expected years of schooling, mean years of schooling, and GNI per capita. Each feature model was trained using linear regression, random forest, and AdaBoost techniques. Hyperparameters were set for each algorithm, and results were evaluated using a 10-fold (k=10) k-fold cross-validation.

#### Model Hyperparameters and Validation

**Figure 2:**
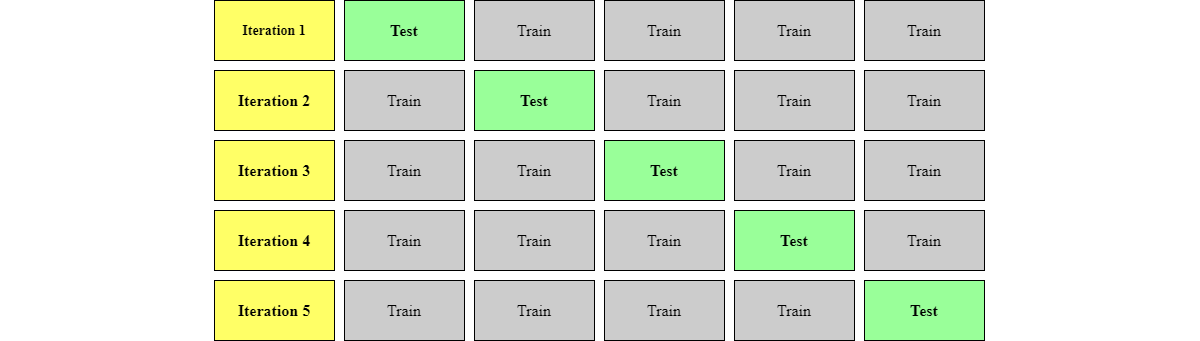
An example of the 5-fold k cross-validation method where k=5. The overall accuracy score is calculated as the mean value of each fold’s accuracy score.

Rather than partitioning the data into training and test sets using the train/test split, this research used k-fold cross-validation. K-fold cross-validation has a single parameter called *k* that represents the number of subsets or “folds” that a data set will be split into, which the user selects. As shown in Figure 2, each fold uses a different grouping of data as the test set, and the process is then repeated *k* number of times (for example, 5 times in Figure 2). It is evaluated by the cross-validation score, which is the mean of all scores from each k-fold subset. K-fold cross-validation provides a more generalizable and less biased performance estimate when working with smaller data sets [76,77]. This is because it maximizes the number of observations that can be used for both training and testing. In other words, a model using cross-validation does not depend on a single train/test split.

Using sklearn, the mean cross-validation score defaults to the scoring metric for the specific algorithm being cross-validated. For each algorithm in this study, the default scoring metric was the coefficient of determination (R^2^). Therefore, the mean cross-validation score computed was the average R^2^ for each algorithm across all k-folds. R^2^ represents the goodness of fit of a regression model and explains how much variance in the dependent variable can be explained by one or more independent variables. It is calculated by dividing the residual sum of squares by the total sum of squares and subtracting the derivation from 1, as follows:

R^2^ = 1 – (residual sum of squares / total sum of squares)

R^2^ was the primary measure under observation in this study. In machine learning, R^2^ is the most informative validation measure with the least interpretive limitations [78].

Table 2 presents the hyperparameters unique to each algorithm. A 10-fold validation was selected for the k-fold cross-validation, which is a generally recommended number of subsets to apply [76,77].

Alongside R^2^, 4 error metrics were also calculated to assess performance. First, MAE provides the average of the absolute error between the predicted values and true values. It is calculated as follows:



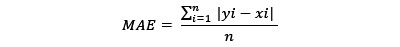



where *y_i_* is the prediction value, *x_i_* is the actual value, and *n* is the number of observations.

Second, MSE measures the average squared difference between the predicted values and true values. It is calculated as follows:



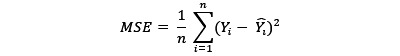



where *n* is the number of data points, *Y_i_* is the actual value, and *Ŷ_i_* is the predicted value.

Third, RMSE calculates the square root of the mean of squared errors of a model. It is calculated as follows:



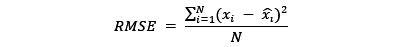



where *i* is the variable *i*, *N* is the number of data points, *x_i_* is the actual value, and *x̂_i_* is the predicted value.

Finally, max error computes the maximum residual error, which captures the worst case error between the predicted value and the true value. It is calculated as follows:



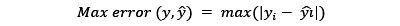



where *ŷ* is the predicted value of the *i*-th sample, and *y_i_* is the corresponding true value.

**Table 2:** Supervised learning model hyperparameters using cross-validation.

Algorithm	Hyperparameters
Basic linear regression	Folds: 10; random state: 1
Random forest	Folds: 10; random state: 1; estimators: 100
AdaBoost	Partitions: 10; estimators: 50; random state: 0

## Results

### Exploratory Data Analysis

Exploratory data analysis was carried out to identify and visualize trends in the data, and to statistically analyze the variables. In 2020, the mean number of COVID-19 cases per million in the sample was 15,880.41, with a median of 6822.98. In 2021, the mean number of COVID-19 cases per million was 64,479.58, with a median of 50,764.73. Table 3 presents the key descriptive statistics of all variables in the study.

**Figure 3:**
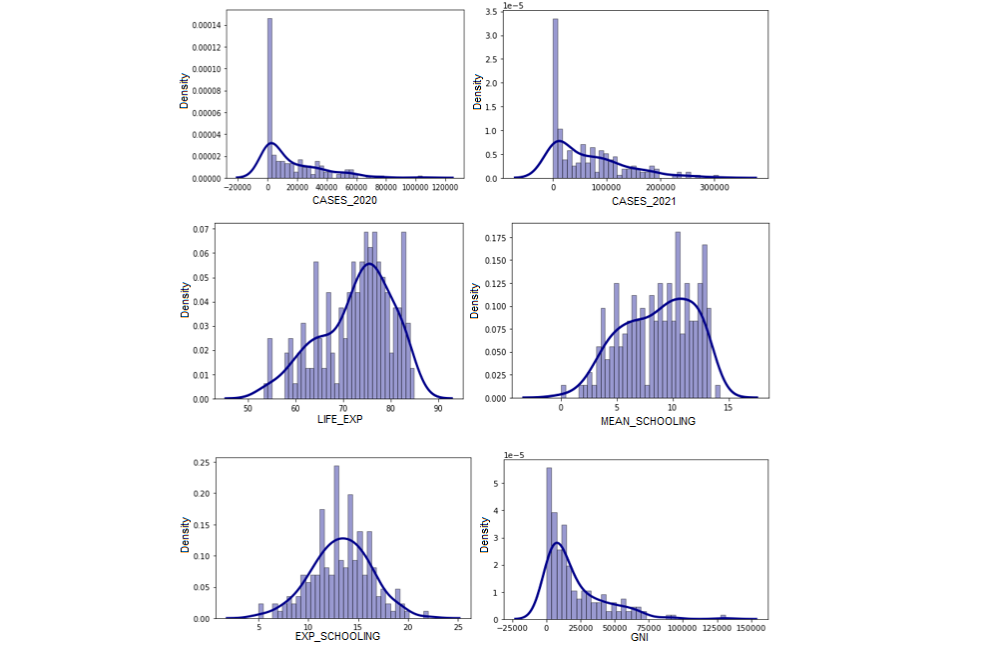
A series of density plots illustrating the distribution of each variable under observation (the target variable). The target variable 2021 COVID-19 cases per million is right-skewed in the sample. Expected years of schooling is the only variable with a normal distribution in the sample. CASES_2020: 2020 COVID-19 cases per million; CASES_2021: 2021 COVID-19 cases per million; EXP_SCHOOLING: expected years of schooling; GNI: gross national income per capita; LIFE_EXP: life expectancy; MEAN_SCHOOLING: mean years of schooling.

Distplots were created to inspect the distribution of all variables. The resulting plots showed that all variables, with the exception of expected years of schooling, were skewed in the sample. The distribution of 2021 COVID-19 prevalence was positively skewed in the sample (see Figure 3). Calculation of the interquartile range revealed that 4 countries (Andorra, Montenegro, Serbia, and Seychelles) were statistical outliers, which had recorded unusually high rates of COVID-19 (>250,000 per million population). The Seychelles recorded the highest prevalence with 217,096.35 cases per million.

**Figure 4:**
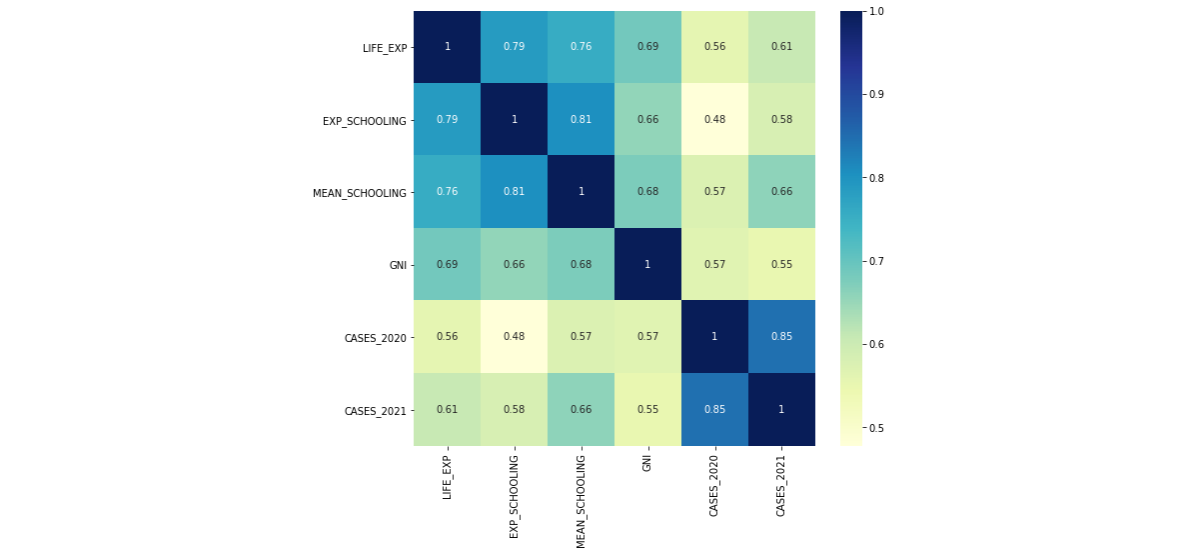
Pearson correlation matrix mapping the correlation between all variables. Results show that all features have a statistical correlation with 2021 COVID-19 cases. CASES_2020: 2020 COVID-19 cases per million; CASES_2021: 2021 COVID-19 cases per million; EXP_SCHOOLING: expected years of schooling; GNI: gross national income per capita; LIFE_EXP: life expectancy; MEAN_SCHOOLING: mean years of schooling.

To investigate the statistical relationship between the features and the target variable, a Pearson correlation matrix was implemented (see Figure 4). All chosen features correlated statistically with 2021 COVID-19 prevalence, with R values ranging from 0.55 to 0.85. Moreover, 2020 COVID-19 cases had the strongest correlation with 2021 case data (R=0.85), followed by mean years of schooling (R=0.66), life expectancy (R=0.61), expected years of schooling (R=0.58), and GNI (R=0.55).

**Table 3:** Statistical measurements (mean and median) of all variables in the study.

Variable	Mean value	Median value
2020 COVID-19 cases per million	15,880.41	6822.98
2021 COVID-19 cases per million	64,479.58	50,764.73
Life expectancy	72.72	74.20
Expected years of schooling	13.31	13.15
Mean years of schooling	8.63	8.95
Gross national income per capita (US$)	20,453.40	13,112.50

### Supervised Learning Models

**Figure 5:**
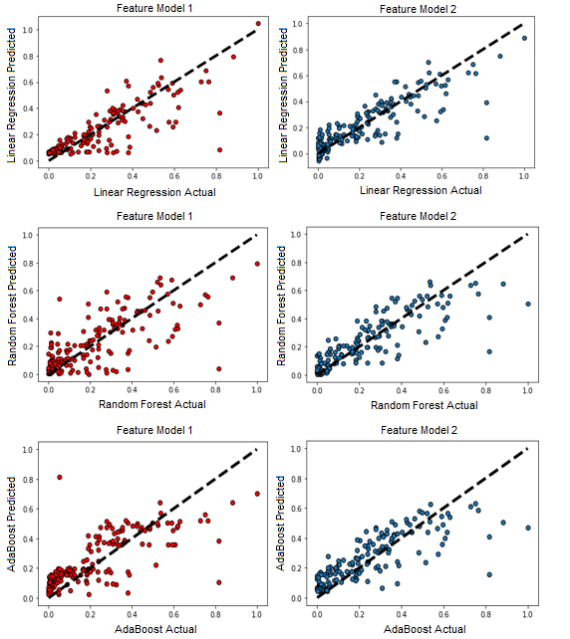
A series of subplots showing the predictive performances of the linear regression, random forest, and AdaBoost algorithms in both Feature Models 1 and 2. Each observation represents a prediction of 2021 COVID-19 cumulative cases per million, with the regression line being the true value. With the addition of Human Development Index metrics, the linear regression algorithm improved from R^2^=0.693 to 0.763. The random forest algorithm improved from R^2^=0.481 to 0.722. The AdaBoost algorithm improved from R^2^=0.454 to 0.679. Data points were calculated using cross_val_predict, which shows the predicted output from each test set within each k fold.

Tables 4 and 5 summarize the performances of all regression algorithms in both feature models, while Figure 5 visualizes their performances. Feature Model 1 was trained to predict 2021 COVID-19 cases per million using 2020 cases per million (n=182). Feature Model 2 was trained to predict 2021 COVID-19 cases per million using 2020 cases per million as well as life expectancy, mean years of schooling, expected years of schooling, and GNI (n=182). Both data sets were divided into 10 folds for cross-validation (k=10).

In Feature Model 1, linear regression was the most accurate learner with a mean R^2^ of 0.693, followed by random forest (0.481) and then AdaBoost (0.454). The variation in performance was considerable, with a 23.9% difference between the most precise and least precise algorithms. In Feature Model 2, the basic linear regression model was also the strongest learner (R^2^=0.762), followed by random forest (0.722) and AdaBoost (0.679). The MAE, MSE, RMSE, and max error statistics of the algorithms were all lower in Feature Model 2 than in Feature Model 1. Feature Model 2 also exhibited closer performances between the algorithms than Feature Model 1, with the strongest learner being 8.4% more precise than the least.

Although it was the best learner on the data in both models, linear regression showed the least improvement with the inclusion of socioeconomic indicators in Feature Model 2 (R^2^ improved by 7%). Additionally, its error statistics did not improve as significantly as those of random forest or AdaBoost. For example, the MAE of linear regression decreased by 0.009 (0.079 in Feature Model 1 and 0.070 in Feature Model 2) compared to decreases of 0.026 in random forest and 0.014 in AdaBoost.

Tables 6 and 7 outline the performance accuracy of each individual fold. The widely varying R^2^ scores indicate that the cross-validation approach used in this study yielded the most reliable results.

**Table 4:** Evaluation of Feature Model 1 using linear regression, random forest, and AdaBoost.

Evaluation measure	Linear regression^a^	Random forest^a^	AdaBoost^a^
R^2^	0.693	0.481	0.454
MAE^b^	0.079	0.096	0.104
MSE^c^	0.014	0.021	0.020
RMSE^d^	0.117	0.143	0.142
Max error	0.315	0.359	0.355

^a^All results were evaluated using k-fold cross-validation (k=10).

^b^MAE: mean absolute error.

^c^MSE: mean squared error.

^d^RMSE: root mean squared error.

**Table 5:** Evaluation of Feature Model 2 using linear regression, random forest, and AdaBoost.

Evaluation measure	Linear regression^a^	Random forest^a^	AdaBoost^a^
R^2^	0.763	0.722	0.679
MAE^b^	0.070	0.070	0.090
MSE^c^	0.011	0.013	0.015
RMSE^d^	0.107	0.114	0.124
Max error	0.265	0.308	0.300

^a^All results were evaluated using k-fold cross-validation (k=10).

^b^MAE: mean absolute error.

^c^MSE: mean squared error.

^d^RMSE: root mean squared error.

**Table 6:** Accuracy for each algorithm’s individual fold (k=10) in Feature Model 1.

Iteration	Linear regression	Random forest	AdaBoost
Fold 1	0.877	0.799	0.759
Fold 2	0.768	0.687	0.342
Fold 3	0.657	0.464	0.584
Fold 4	0.803	0.530	0.629
Fold 5	0.747	0.153	-0.696
Fold 6	0.733	0.553	0.766
Fold 7	0.804	0.628	0.652
Fold 8	0.035	-0.287	0.083
Fold 9	0.767	0.627	0.696
Fold 10	0.742	0.657	0.722

**Table 7:** Accuracy for each algorithm’s individual fold (k=10) in Feature Model 2.

Iteration	Linear regression	Random forest	AdaBoost
Fold 1	0.774	0.796	0.679
Fold 2	0.595	0.457	0.485
Fold 3	0.946	0.907	0.882
Fold 4	0.602	0.622	0.551
Fold 5	0.833	0.869	0.824
Fold 6	0.780	0.776	0.720
Fold 7	0.627	0.636	0.626
Fold 8	0.850	0.659	0.536
Fold 9	0.780	0.794	0.851
Fold 10	0.844	0.594	0.629

## Discussion

### Principal Findings

Results from exploratory data analysis yielded a number of interesting insights. First, the positively skewed distribution of 2021 COVID-19 cases resulted in a mean greater than the median in the sample. In the 182 countries sampled, COVID-19 prevalence was asymmetrical and revealed that a minority of countries recorded very high case numbers. Second, the distribution of 2020 COVID-19 cases was positively skewed and similar visually to the 2021 distribution. This shows that the trajectory of the virus in the sample was relatively consistent in 2020 and 2021 in terms of cumulative reported cases. Third, the 4 outlier countries identified shared an interesting pattern; all had higher than average life expectancy, mean years of schooling, and GNI compared with the means in the sample. This indicates that the outliers can be considered above average socioeconomically. Finally, all HDI metrics correlated positively with COVID-19 cases per million, which points to an important statistical relationship between socioeconomic status and COVID-19 prevalence. Education (expected/mean years) shared the highest correlation, followed by life expectancy and then GNI. This correlation is noteworthy and highlights the unique nature of the COVID-19 pandemic. Typically, lower socioeconomic status is associated with poorer health outcomes, but the results from this study suggest that countries with higher socioeconomic status recorded higher rates of COVID-19 in 2021. This could be because more developed countries tend to have older populations, as well as higher prevalence of known COVID-19 clinical risk factors, such as diabetes and cardiovascular disease [79].

The results from machine learning analysis suggest that 2021 COVID-19 prevalence could be predicted with a reasonable degree of accuracy using the previous year’s prevalence rates and the socioeconomic indicators of life expectancy, mean years of schooling, expected years of schooling, and GNI per capita. With socioeconomic indicators included, the R^2^ of each learning algorithm was higher than that when trained on only 2020 COVID-19 data, and the error statistics were lower. Including the HDI indices as predictors alongside the previous year’s COVID-19 cases in each country improved the predictive accuracy of 2021 cases by an average of 18% across the 3 chosen algorithms. Given that predictive algorithms can struggle with smaller data sets [59], the results of this study (n=182) are noteworthy.

The linear regression algorithm was the strongest learner on the data, but also showed the least improvement (7% increase in mean cross-validation) once the HDI metrics were added. Given that the other algorithms improved considerably when HDI indices were added, this result represents an interesting outlier. The varying performances between the algorithms may be due to the statistically linear relationships between the variables (as discovered in the Pearson correlation matrix in Figure 4). Despite the strong correlation between 2021 COVID-19 cumulative cases per million and case data from the previous year (R=0.84), Feature Model 1 did not make accurate predictions using random forest or AdaBoost. Unlike linear regression models, which excel at fitting to data where linear correlation exists, decision tree algorithms like random forest and AdaBoost may perform more effectively with nonlinear data [80,81]. Lastly, the widely varying performance of each k-fold iteration justified the use of cross-validation to evaluate the models. In Feature Model 2, for example, the highest scoring fold of the linear regression algorithm had a result of 94.6, a highly accurate R^2^ result. However, the lowest scoring fold had an R^2^ of 59.5. The cross-validation R^2^ score of 76.3 was therefore the most reliable score for the data set.

### Follow-Up Analysis

Following the primary analysis, 4 follow-up analyses were conducted. First, Feature Model 2 was trained again without 2020 COVID-19 case data as a feature to analyze how well the HDI metrics could predict COVID-19 cases alone. Without the previous year’s case data, the accuracy was low (R^2^=0.438 for the best performing algorithm, which was again linear regression). This result highlights the significant importance of 2020 case data in predicting the following year’s COVID-19 prevalence. Second, Feature Model 2 was trained again using 1 HDI metric at a time to analyze which was the most important for the prediction of COVID-19 cases. The results showed that expected years of schooling and mean years of schooling had the highest scores (R^2^=0.755 for each), followed by life expectancy (R^2^=0.739) and then GNI (R^2^=0.712). This suggests that education was the most predictive socioeconomic indicator (the education HDI metrics were also the most statistically correlative). However, the results also showed that using all HDI indices is more effective than using them separately for COVID-19 case prediction in this data set. The third follow-up experiment removed the 4 previously identified outlier countries (Andorra, Montenegro, Serbia, and Seychelles) from the data set and implemented both feature models again, using the same cross-validation method as the initial analysis. This yielded interesting results (see Tables 8 and 9). Most notably, random forest became the strongest learner in Feature Model 2 (R^2^=0.777). Despite being generally less sensitive to outliers [82], random forest benefitted from outlier removal in this data set. Removing outliers also reduced the gap in performance between the algorithms. With outliers included, Feature Model 1 displayed a 23.9% difference between the best and worst performing algorithms, and with outliers removed, this difference reduced to 19.5%. This reduction was more apparent in Feature Model 2, with just a 2.1% difference between the best and worst performing algorithms with outliers removed (compared with an 8.4% difference in the original sample with outliers included). However, the results indicated that removing the outliers did not significantly improve overall predictive accuracy.

The fourth follow-up experiment sought to compare socioeconomic status as a COVID-19 predictor with a selection of other COVID-19 risk factors. Subsequently, each country’s median age, population density (individuals per square kilometer), and percentage of vaccinated individuals were sourced and added to the data set. Each of these variables has been shown to predict COVID-19 prevalence in certain samples [83-85]. Most of the required data were also available in the OurWorldInData database, though a small number of entries had to be sourced from Worldometers and IndexMundi [86,87].

When Feature Model 2 was trained again using these new metrics alongside 2020 case data, predictive accuracy dropped to an average of 0.649 across all 3 algorithms. Using these new features, the most accurate algorithm was 10% less accurate than the most accurate learner in the model with socioeconomic features (see Table 10). This is a significant finding, which suggests that socioeconomic status was more effective in predicting 2021 cumulative cases than a country’s median age, population density, and vaccination uptake, highlighting its unique importance as a nonclinical predictor of COVID-19 in the sample of countries.

**Table 8:** Feature Model 1 comparison (outliers included versus excluded).

Algorithm	Mean R^2^ in the sample with outliers included (n=182)	Mean R^2^ in the sample with outliers excluded (n=178)
Linear regression	0.693	0.689
Random forest	0.481	0.493
AdaBoost	0.454	0.494

**Table 9:** Feature Model 2 comparison (outliers included versus excluded).

Algorithm	Mean R^2^ in the sample with outliers included (n=182)	Mean R^2^ in the sample with outliers excluded (n=178)
Linear regression	0.763	0.754
Random forest	0.722	0.777
AdaBoost	0.679	0.733

**Table 10:** Feature Model 2 performance comparison of socioeconomic metrics versus other risk factors using linear regression.

Measure	Feature Model 2 with HDI^a^ indicators	Feature Model 2 with population density, median age, and vaccination uptake
R^2^	0.763	0.661
MAE^b^	0.070	0.075
MSE^c^	0.011	0.016
RMSE^d^	0.107	0.128
Max error	0.265	0.312

^a^HDI: Human Development Index.

^b^MAE: mean absolute error.

^c^MSE: mean squared error.

^d^RMSE: root mean squared error.

### Significance of the Results

In order to put the machine learning results of this study into perspective, we compared the best performing algorithm (R^2^=0.763) with similar machine learning COVID-19 case predictions. Overall, it fits within the accepted range of COVID-19 predictive modeling studies in the systematic review mentioned earlier, which ranged from 0.64 to 1 [64]. Results from this study align with the findings from another study that attempted to predict COVID-19 cumulative cases in 3109 counties in the United States using a multilayer perceptron neural network. In this previous study, the socioeconomic indicator of median household income ranked fifth among 57 clinical and nonclinical predictor variables of COVID-19 prevalence [88]. Studies, such as this, portray the importance of socioeconomic indicators as determinants of COVID-19 prevalence rates, which further supports the use of HDI in this study to more accurately and precisely predict COVID-19 prevalence in 2021.

This research has a number of implications. First, it showcases the utility of combining statistical and machine learning approaches in pandemic research. Although statistical tests can determine correlations between variables, they cannot provide specific predictions of the target variable. Each method thus addresses a shortcoming of the other. Second, this study indicates that socioeconomic status is an important variable to consider in future epidemiological modeling, and reveals the complex social nature of the COVID-19 pandemic. Socioeconomic status was a better predictor of COVID-19 prevalence than median age, population density, and vaccination uptake. Third, the accuracy of these results in a multicountry sample is noteworthy. Owing to the data taken from 182 countries, this research suggests that socioeconomic status can be considered a “global risk factor” rather than a country-specific factor [38]. This will support evidence-based policy and interventions by decision makers. Fourth, the results indicate that although socioeconomic factors aid in the prediction of COVID-19, there could be other important factors that could further optimize prediction. Finally, the importance of historically reported COVID-19 case data cannot be understated in attempting to predict future COVID-19 prevalence. The 2020 COVID-19 case data correlated strongly with 2021 COVID-19 case data and could be considered the most important machine learning feature.

### Limitations

As with all research studies, there are inherent limitations in this study. First, when analyzing COVID-19 cross-nationally, it must be noted that some countries have underreported their number of cases more than others for reasons, such as limited testing capacity [89]. Second, there are other socioeconomic factors that the HDI does not account for, including levels of financial inequality, social exclusion, or discrimination within countries [90]. These factors are worthy of inclusion in future research to assess their impact. Third, national COVID-19 prevalence rates give an overall measure of how severely a country is impacted, which is suitable for cross-country research, but they do not capture the full complexity of transmission patterns within each country. It is recommended that further research be conducted at the regional and municipal levels to assist pandemic forecasting. Lastly, it can be challenging to train reliable machine learning models using small data sets [59]. Cross-validation was used to address this limitation, as it maximizes the data set and minimizes the potential bias of a traditional partitioning approach.

### Conclusions

A better understanding of population-level predictors is of crucial importance to better understand and respond to public health crises caused by COVID-19 [91]. This study contributes to the growing corpus of COVID-19 predictive modeling research by showing that socioeconomic status is an important nonclinical risk factor. Using HDI and historical case rates, it was observed that 2021 cross-national COVID-19 cumulative cases could be predicted with a reasonable degree of accuracy. Although COVID-19 represents a long-term challenge for the global society, the data-driven approach of machine learning will continue to support decision makers in understanding the pandemic, formulating response strategies, and predicting future outcomes [92].

## Abbreviations

GNI: gross national income
HDI: Human Development Index
MAE: mean absolute error
MSE: mean squared error
RMSE: root mean squared error
SIR: susceptible, infected, recovered
UNDP: United Nations Development Programme
